# Function, chance and purpose in the biosphere: a critical examination of the Darwinized Gaia hypothesis

**DOI:** 10.1098/rstb.2024.0099

**Published:** 2025-08-07

**Authors:** Margarida Hermida, Samir Okasha

**Affiliations:** ^1^ King's College London, London, UK; ^2^ Department of Philosophy, University of Bristol, Bristol, UK

**Keywords:** biosphere, Darwinized Gaia, evolution, natural selection

## Abstract

The original Gaia hypothesis purports to explain the long-term maintenance of the Earth’s habitability by proposing that the biosphere has evolved homeostatic control of environmental parameters crucial to its survival. This idea was criticized for being incompatible with core Darwinian requirements for evolution by natural selection, since the biosphere is not part of a population of entities with variation, reproduction and heredity. Recently, however, some authors have defended a ‘Darwinized’ version of the Gaia hypothesis. Proponents of Darwinized Gaia argue that there can be natural selection for persistence alone, with no need for reproduction or competition, and that a single lineage can undergo a process of sequential selection over time. This article examines these ideas in the light of two important distinctions in evolutionary biology: adaptation versus by-product, and transformational versus variational explanation. Despite these new interesting ideas, however, the Darwinized Gaia hypothesis still cannot overcome the main objections from evolutionary biology.

This article is part of the discussion meeting issue ‘Chance and purpose in the evolution of biospheres’.

## 1. Introduction

Conditions on Earth have been suitable for the presence of life for the past 4 billion years. Evidence suggests that the very fact that the Earth is inhabited has contributed to the long-term persistence of habitable conditions [1]. The composition of the Earth’s atmosphere, the presence of liquid water on the surface and relatively stable temperature ranges despite a steady increase in solar radiation over time are all thought to partly depend on biotic, in addition to abiotic processes. The Gaia hypothesis, introduced by Lovelock & Margulis [2], suggests that an explanation for the maintenance of habitable conditions on Earth is the homeostatic control of environmental parameters by the biosphere itself. In their words, life ‘acquired control of the planetary environment’ soon after it originated, and ‘this homeostasis by and for the biosphere has persisted ever since’ [2, p. 2].

Two clarifications are in order. First, the fact that the continued presence of life both depends on, and partly contributes to, the continued habitability of Earth does not by itself imply that there is a homeostatic mechanism at play. The long-term maintenance of habitable conditions could be no more than a happy by-product of biotic processes. Tyler Volk accepts that Gaia is simply the result of by-products of the metabolic activities of various organisms, and that organisms do not ‘construct the environment for their own benefit’ [3, p. 34], but he still wants to count this as a development of the Gaia hypothesis. In our view, this amounts to the rejection of the Gaia theory, rather than a development of it. It is not really under dispute that the existence of life contributes to the continued habitability of Earth; the question is whether this is because life *homeostatically regulates* environmental parameters for its own benefit, or whether it is simply a lucky by-product.

Second, it is important to establish what exactly is the phenomenon that the Gaia hypothesis purportedly explains. It cannot simply be the difference in habitability between Earth and two uninhabited nearby rocky planets, Venus and Mars, since, at least for the early stages of the solar system, that difference can be explained in terms of differences the solar radiation, size and initial atmospheric conditions of each planet. It is also not stability *per se* that requires an explanation; on the contrary, we should expect all planets that receive solar radiation and emit heat to reach a stable steady state that reflects the various inputs and outputs of the system, and this often involves chemical cycling processes, driven by the energy that flows through the system. Since, on an inhabited planet, biotic processes contribute to chemical cycling, they too become part of the planet’s steady state and therefore contribute to its stability. None of this requires postulating homeostatic mechanisms. If there is anything that requires a ‘Gaian’ explanation, it is the long-term persistence of habitable conditions on Earth. The Gaia hypothesis proposes that the ‘control’ exerted by the biosphere is not a mere by-product of the existence of biotic processes, in the same way that some abiotic processes are often said to ‘control’ other abiotic processes (e.g. [4]), but it is the result of evolved homeostatic mechanisms that promote the long-term survival of the biosphere.

The idea of homeostatic mechanisms at the level of the whole biosphere is clearly meant to be analogous to the presence of homeostatic mechanisms in living systems. Organisms have evolved various homeostatic mechanisms that allow them to maintain certain parameters (such as pH, chemical composition, temperature, etc.) within restricted ranges that are suitable for their functioning. Like any other traits of living organisms, homeostatic mechanisms result from an evolutionary process involving: (i) variation in traits within a population, which (ii) correlate with fitness differences, and (iii) parent’s traits tend to be inherited by offspring—these are generally recognized as the minimal conditions for evolution by natural selection [5,6]. At first sight, neither of these conditions is present in the Gaia case: there is no population of entities and therefore no variation, no reproduction and indeed no selection. It is hard to see how a Darwinian process could possibly take place in this case. Thus, the traditional neo-Darwinian critique of the Gaia hypothesis holds that it is simply not possible for a biosphere-level entity to evolve homeostatic mechanisms, or any other adaptations, because there is no selection at that level. It is of course unproblematic for natural selection to operate on the components of the biosphere, such as organisms or species; however, selection at these levels would tend to favour traits that benefit the entities undergoing selection, regardless of their effects on other entities or on the biosphere as a whole, as opposed to traits that benefit the whole biosphere. For these reasons, the Gaia hypothesis was widely rejected as a scientific hypothesis.

More recently, however, Doolittle and others have proposed ‘Darwinized’ versions of the Gaia hypothesis, which supposedly overcome the standard Darwinian objections and specifically attempt to place it within a Darwinian framework. They argue that natural selection does not necessarily involve competition between reproducing individuals, and instead merely differential survival may be sufficient for natural selection [7–9], that ‘sequential selection’ may occur within a single entity over time [10] and that the relevant units of selection may be not be organisms or biological taxa at all, but the biogeochemical cycles or feedback cycles themselves, an idea captured in the ITSNTS (it's the song not the singer) slogan [8,9,11,12].

Our aim in this article is to critically scrutinize the case for a Darwinized Gaia hypothesis, drawing on philosophical considerations to do with the logic of Darwinian explanation. The outline of the paper is organized as follows: §2 discusses the distinction between function and by-product in evolutionary biology and how this distinction becomes blurred in the Darwinized Gaia hypothesis; §3 discusses two evolutionary mechanisms that have been invoked by proponents of Gaia: persistence selection and sequential selection; §4 asks whether these forms of selection are properly Darwinian, understood as involving a variational, rather than transformational explanation; §5 is a brief conclusion.

## 2. The function versus by-product distinction

At the heart of the debate over the scientific status of the Gaia hypothesis is the distinction between function and incidental by-product. The traditional neo-Darwinian objection to the Gaia hypothesis was that even if biotic processes do contribute to Earth’s continued habitability, this is not their evolved function but merely a fortuitous side-effect, or by-product. This can equally be expressed by saying that the biosphere, or the Earth-life system, is not itself an adapted unit—for a biological entity only counts as an adapted unit if it exhibits traits that evolved because of the benefit they bring at the level of the whole unit. Proponents of Darwinized Gaia are of course aware of this objection but hope to circumvent it.

To assess the debate, it is worth attending briefly to the function/by-product distinction itself. Though it has been marked using various terms, this distinction is central to the practice of evolutionary biology and is implicitly appealed to whenever biologists discuss the adaptive significance (purpose, function) of an organismic trait. Take for example, the bird of paradise’s courtship dance. The usual evolutionary story has it that the function of this dance is to attract mates, meaning that that is why natural selection led it to evolve and/or be maintained in the population. The dance also attracts predators, however, this is not part of its function; it is just something that the dance happens to do. That is, the dance evolved in spite of, not because of, its predator-attracting effect.

Notice that in this example, the by-product is manifest at the same level of the biological hierarchy as the trait of which it is a by-product. That is, the courtship dance is a trait of the bird (an individual organism), and attracting a predator is something that happens to the bird itself. But, in other cases, by-products may be manifest at other hierarchical levels. The earthworm’s burrowing aerates the soil, which increases the soil’s microbial biomass and thus leads to greater plant diversity in the ecological community. Here, the by-product of an organismic activity, burrowing, is an effect manifest at the level of the community to which the burrowing earthworms belong.

In most contexts, the distinction between function and by-product is conceptually clear and unproblematic. It may not always be clear how to *apply* the distinction, that is, how to tell whether a particular effect is a function or a by-product, but this is an epistemological limitation. If we had complete knowledge of the course of evolutionary history, including the ecological reasons why some genes and traits were selected over others, then in principle it should be straightforward to determine what is a function and what is a by-product. However, there is one type of case in which the function/by-product distinction becomes blurred, and not because of epistemological limitations. This type of case, we suggest, is particularly relevant to the Darwinized Gaia debate.

Consider a hierarchically structured biological system consisting of smaller units (individuals) nested within larger units (groups). Suppose that both individuals and groups engage in reproduction, understood very broadly as ‘making more of themselves’, and that the turnover rate of individuals is faster than that of groups. Suppose that selection takes place at the individual level, leading to the evolution of an individual trait which has, as a by-product, an effect on one or more group attributes. A second selection process acts at the group level, over a longer timescale, in which the group-level trait that is selected for is, in part or in whole, a downstream effect of the individual-level selection. Now let us ask ‘is the group-level trait a selected function or an incidental by-product?’ It is unclear what the answer is, even if all relevant empirical facts are assumed to be known.

As an illustrative, if hypothetical, example, consider a clade containing a number of sister species. Suppose that within some of these species, individual-level selection favours natal dispersal, perhaps to minimize inbreeding. As a result, these species come to have a wider geographical range than others in the clade. Now suppose that a process of species-level selection takes place (*sensu* [13]), and that the species trait subject to selection is geographical range. For example, the wider a species range, the less prone it may be to extinction or the more prone to bud off daughter species (speciation may be regarded as ‘reproduction’ for species). Thus, over macroevolutionary time, the average geographical range of species in the clade will increase. Is the trait ‘wide geographical range’ then a function or a by-product? A case can be made either way. Wide range arises as a by-product of selection at the individual level, but it is then subject to a process of selection at the species level.

This issue has never been explicitly addressed in either the biological or philosophical literatures, so far as we know. However, a related issue was discussed in the debate over species selection in the 1980s. A number of authors argued for a distinction between ‘genuine’ species selection and what they called ‘species sorting’ [14,15]. The intuitive idea was that differential survival/speciation might be evidence of a selection process acting at the species level, but might also be a side-effect of lower-level processes ‘filtering up’ the biological hierarchy. To illustrate the latter possibility, suppose that grey squirrels are better foragers than red squirrels, who are eventually driven to extinction. This then is a case of differential species survival that (we may presume) is not due to chance. But it seems wrong to attribute it to a causal process acting at the level of the whole species. The survival of the grey species, and the extinction of the red, is just a consequence of the fact that individual grey squirrels were competitively superior to their red counterparts. That is, selection at the individual level favoured grey over red squirrels, and the differential species survival was a side effect. This then counts as species sorting, not selection.

Although intuitive, the selection/sorting distinction, whether at the species or other level, is not easy to make precise. The distinction cannot simply turn on whether individual-level events and processes, including selection, have a causal influence on the higher-level entities to which the individuals belong, for this will presumably always be true. In any hierarchically structured system, attributes of the larger units (e.g. organisms) will systematically depend on the smaller units (e.g. cells) that compose them. The dependence may be more complex in some cases than others, but will presumably always be there. (This is related to what philosophers call ‘mereological supervenience’, or part-whole determination.) And, it will be true of fitness components, such as the probability of surviving or avoiding extinction, just as much as any other phenotypic attributes.

How then should the distinction between selection and sorting, in the context of a hierarchically structured system, be unpacked? A suggestion due to Damuth & Heisler [16], developed by [17,18], shows the way. Suppose that there is differential survival/reproduction of the groups. This is equivalent to saying that there is *trait–fitness covariance* at the group level, that is, some group trait covaries with the (realized) group fitness (as measured by group persistence or reproduction). Thus, in the squirrel example, the species trait ‘proportion of grey squirrels’ (which takes the value 1 for the grey species, 0 for the red) covaries with species persistence. The crucial question, then, is not whether the group trait, and the group fitness, are themselves causally determined by individual-level events and processes (including selection); they always will be. Rather, the question is whether the covariance between group trait and group fitness is attributable to, that is, causally explained by, individual-level selection or not.

In the squirrel example, the answer to this question is yes. It is because selection at the individual level favours grey squirrels over red, that the species trait ‘proportion of grey squirrels’ covaries positively with species persistence. In contrast, in a bona fide case of species selection, this will not be so. Take for example the hypothesis that sexual reproduction in animals has been maintained by selection against parthenogenetic species who lack the genetic variation needed to adapt to environmental shocks. If this hypothesis is true, then the species trait ‘contains sexually reproducing organisms’ covaries positively with a component of species fitness (i.e. persistence), but not because of selection at the individual level. It remains the case, though, that this species trait causally depends on the properties of the individuals in the species, and similarly for species fitness.

We suggest that this way of capturing the selection versus sorting distinction also deals with the ‘problem cases’ for the adaptation versus by-product distinction. So long as there is a true selection process at the higher level, that is, a causal influence of trait on fitness at that level, then the higher-level trait that is selected for has a function at that level, even if it originated as a by-product of individual-level selection. Thus, in our previous example, having a wide geographic range does indeed have a function at the species level (such as buffering the species against extinction). Even though selection on the individuals within a species, for natal dispersal, causally affects a species’ geographic range, the key point is that individual-level selection does not causally explain the covariance between geographic range and species fitness within the clade. Thus, being a by-product of lower-level selection does not automatically prevent a higher-level trait from being functional at the higher level.

What has this got to do with Gaia? One of the arguments made by proponents of the Darwinized Gaia hypothesis is that even though the Earth does not reproduce, it may nonetheless have been subjected to ‘persistence selection’, or ‘selection acting purely on survival’. Granting (for now) that this counts as a bona fide form of natural selection, the question arises whether this observation suffices to rebut the traditional neo-Darwinian objection to the Gaia hypothesis, which to recall was that although biotic processes may contribute to the Earth’s habitability, this is not their function but just a by-product.

If what we have said about function versus by-product in hierarchically structured systems is correct, we can say the following. Even though the biotic processes in question originally came into existence via lower-level causal processes, including (presumably) natural selection at the individual level, this does not automatically preclude them from being subject to a selection process at a higher level, in which case they would indeed be functional at that level. For to repeat, the mere fact that a trait of a higher-level entity arises from lower-level selection is not to the point; covariance with components of fitness is what matters. So, in principle, the Darwinized Gaia response to the ‘fortuitous by-product’ objection could succeed; however, this all presumes that persistence selection is indeed a genuine mode of natural selection. We turn next to this question.

## 3. Persistence selection and sequential selection

Early attempts to reconcile Darwinian evolution with the Gaia hypothesis focused on the development of models, such as Daisyworld, where natural selection at the level of individual organisms is coupled with global temperature regulation [19]. These models show that it is possible for there to be negative feedback between biotic and environmental parameters, which results in stability, but under different circumstances, it is also possible for positive feedback to emerge, with disruptive effects [20]. These models also do not propose a selection mechanism at the biosphere level, so whatever feedbacks occur are mere side-effects of selection at the individual level, rather than biosphere-level adaptations.

### (a) Persistence selection

Doolittle [7] has proposed natural selection through survival alone as a mechanism for evolution of the biosphere. Natural selection is commonly thought to require a population of reproducing entities, but Doolittle [7] suggests that natural selection would also occur in a population of potentially immortal entities that do not reproduce and do not directly compete for space or other resources, but simply have differential survival. Within such a population, whether an individual dies and another survives may be the result of pure chance, but it may also be the case that some individuals happen to acquire mutations that increase their probability of survival. Since individuals differ in properties that correlate with their survival, a Darwinian selection mechanism is operative: individuals that lack persistence-enhancing mutations (or have acquired deleterious ones) are more likely to be removed by natural selection, whereas individuals with persistence-enhancing mutations are more likely to survive for longer. The persistence-enhancing mutations could be the by-product of selection at a lower level, which then provides the variation for selection at the higher level.

Although Godfrey–Smith argues that selection without reproduction is ‘a pale analogue of the Darwinian process’ [6, p. 104], there is reason to count this process as bona fide Darwinian, insofar as it presents a selectionist explanation of the fact that individuals bearing persistence-enhancing mutations in the population will become more frequent in the population over time. Doolittle’s idea seems to stand up to scrutiny—if some individuals have mutations that tend to increase survival, then selection through survival alone will indeed lead to these individuals being over-represented in the population at later times, and their survival at these later times is increasingly more likely to be explained not merely by their having survived chance extinction events, but also due to the mutations they have acquired by chance, which increased their probability of surviving those events. This can be considered a form of natural selection even though it does not involve reproduction, and it may be a plausible mechanism of natural selection among lineages. Doolittle [7] also claims that these ‘mutations’ can be adaptations, even though they arise through selection at lower levels. Although, in the absence of reproduction, these higher-level traits are by-products of selection on lower-level entities, it is possible to make the case that they are adaptations of the higher-level entity if there is selection at the higher level since, in that case, the higher-level trait can be said to have a function at that level. In other words, its maintenance is explained by selection at the higher level, even though its origin is not.

A more significant problem arises, however, when moving from considering a population of non-reproducing things to considering only one. Doolittle [7] argues that, if a surviving lineage at a later time is more likely to bear persistence-enhancing adaptations, then this is also the case even if there was only one such lineage to begin with. But what legitimates the idea that those chance mutations are ‘adaptations’ in the first place is the occurrence of natural selection at the higher level, which is responsible for increasing their frequency in the population over time. In the absence of a population, there is no natural selection at the higher level; therefore, there are no higher-level adaptations, either.

A different approach consists in thinking of *biogeochemical cycles* themselves as units of selection [9]. It is well understood that organisms in complex ecosystems are highly interdependent, and often the waste product of an organism can be recycled by other organisms. These kinds of situations can generate cycles wherein various organisms participate by carrying out a single biochemical reaction. Once one of these cycles is in place, it does not really matter which organisms carry out which functions—phylogenetically diverse groups of organisms can be recruited from the environment to carry out those same biochemical steps, perpetuating the cycle—an idea captured by the slogan ‘it’s the song, not the singer’ [9,11,21]. The organisms that participate in these cycles would evolve by natural selection, and persistence selection could take place among populations comprising different cycles. This approach goes some way towards addressing the need for a population of things among which natural selection can operate, since there is more than one biogeochemical cycle. It also makes explicit the requirement for the cycle-interaction component of fitness to make a difference to gene frequencies, although these genes may be found scattered throughout many different taxonomic groups of organisms (the ‘singers’) that compose the same metabolic guilds. There are, however, significant problems with identifying and individuating cycles, such that it is hard to tell which instances should be considered variants of the same cycle (and therefore, potentially undergoing persistence selection), and which ones should be considered cooperative—and therefore, not in competition [9]. Furthermore, although this mechanism may contribute to long-term environmental stability, Doolittle [9] argues that Gaian adaptations require thinking of these cycles as forming an even more inclusive single unit, which comprises the whole biosphere. But this raises, again, the problem of uniqueness—this entity no longer seems to be part of a population of entities that can undergo persistence selection.

A possible solution to the problem of uniqueness is to postulate a population of entities. For example, Doolittle [22] suggests that there may have been clade selection among different life clades in the early Earth, and Hermida [23] argues that there may be natural selection among independently originated life clades in the universe. If persistence selection is accepted, then these are indeed relatively unproblematic forms of natural selection.

It is possible that Earth-life, the clade to which all extant life on Earth belongs, was the most successful of an initial population [22]. However, if there were multiple competing life clades on Earth, the other ones must have gone extinct very early in evolutionary history, whereas most of the traits of Earth-life that Gaia theory aims to explain only emerged much later, so cannot be explained by clade selection on the early Earth. It may well be the case that, as Doolittle [24] argues, clade selection is still taking place within the Earth-life clade comprising LUCA and all its descendants, but this is distinct from selection at the life-clade level, and cannot explain adaptations at that level. Furthermore, unlike the case of clone selection in a chemostat [25], there is little reason to think that selection among sub-clades of Earth-life should lead to a single species or sub-clade becoming the ancestor of all species left alive at some future time’ [24, p. 148] (which, in any case, would still constitute the persistence of the Earth-life clade). On the contrary, insofar as ecological and metabolic diversity are associated with phylogenetic diversity, such a development would significantly reduce the resilience of the biosphere.

It is also possible that life clades with survival-enhancing features may become more common in the universe over time [23]. Selection on a putative population comprising all life in the universe may indeed explain the increased frequency over time of life clades with survival-enhancing features—which may even be adaptations. It cannot, however, explain the evolutionary processes that gave rise to them in the first place in a particular instance. In the absence of reproduction, higher-level selection can only account for the maintenance, or increase in frequency, of a trait in a population, but not its emergence, which must be explained in terms of lower-level selection.

### (b) Sequential selection

Lenton *et al*. [10] have proposed a distinct mechanism for the evolution of environmental regulation at the biosphere level: sequential selection for stability. The idea is drawn from cybernetics, and was inspired by Ashby’s [26] ‘homeostat’, a device which controlled several variables, maintaining a stable state until one of the variables deviated from a predefined range, whereupon it would randomly rewire its connections until a new stable state was found. The notion can be applied to life on Earth through sequential selection. Life has some effects on the environment; these effects can either be positive or negative for life. If the effects are positive, this stimulates growth and life thrives. Eventually, the environmental effects may turn out to become environment-degrading, producing a negative feedback on growth and maintaining a stable state over time. Alternatively, negative environmental effects may be so bad that the system approaches the bounds of habitability (but falling short of complete extinction). This resets the system, and eventually, a new stable state is found. This mechanism is, as the authors admit, satisficing rather than optimizing—regulatory mechanisms cannot be refined over time [10, p. 639]; stable states of the systems can only persist or be reset, and nothing guarantees that the next stable state will be more favourable for life than the previous one.

Arthur & Nicholson [27] propose a ratcheting mechanism based on a combination of sequential selection and ‘memory’, whereby later resets of the system draw from a more diverse pool of organisms than earlier ones, which makes it easier for persistence enhancing feedback loops to arise [27]. Although resets carry a non-negligible risk of extinction, they also provide new evolutionary opportunities and lead to higher biomass and environmental stability, when compared to an alternative history of life that did not include these resets [28].

These ideas all seem to make sense, but it is unclear whether sequential selection is relevantly similar to Darwinian natural selection, or even whether it is selection in any meaningful sense. It is unsurprising that complex systems tend to spend more time in stable states than in unstable states and, upon the collapse of a stable state, will tend to reach a new stable state, which may be different from the previous one. However, each new stable state is not selected from among several different states; at most, the system resets select among different ways that a system could be—but this is very different from Darwinian natural selection. On the other hand, the suggestion that resets drawing from a greater biodiversity pool should lead to higher stability is reminiscent of the idea that higher biodiversity and complexity increases ecosystem resilience; however, the connection between these factors is not quite as straightforward as it might seem. In any case, if later states of the biosphere are more resilient than earlier ones—which is plausible—this does not show that there has been selection for resilience, much less that the biosphere has evolved adaptations for control of environmental parameters.

## 4. Is it really Darwinian?

One way to assess the viability of Darwinizing Gaia is to ask whether sequential selection on a single lineage counts as a genuinely Darwinian process. Now opinions differ about what features a process must have in order to count as ‘genuinely Darwinian’; some authors hold that this is a matter of degree, not a yes/no matter [6]. But we can make progress by focusing on a crucial feature of Darwinian explanations, namely the fact that they are variational rather than transformational *sensu* Lewontin [29]; and a logical consequence of this, which is that they explain facts about populations rather than individuals. We suggest that a process only counts as Darwinian, or selectionist, if scientific explanations that invoke that process are variational in character and thus explain population-level facts.

The original point of the variational/transformational contrast was to highlight the logical difference between the Darwinian and Lamarckian theories of adaptation. Lamarck’s posited mechanism—use and disuse of organs—explains how individual organisms become adapted to their environment within their lifetime: they are ‘transformed’ from being non-adapted to well-adapted. In contrast, Darwin’s posited mechanism—selection on heritable variation—explains how populations of organisms come to be adapted over many generations. Thus, despite the long tradition of pitting Darwin against Lamarck, in a sense, their theories are not opposed since they explain different phenomena.

This last point is nicely brought out by an example of Sober [30]. Suppose we observe that the schoolchildren in a particular class are all unusually good at music. What might explain this? One possible answer is to point to the intensive musical training and encouragement that each child in the class received from an early age. A second possible answer is to note that only children who pass a difficult music exam are admitted to the class in the first place, i.e. there is a selective filter that determines entry to the class. These two answers are not in conflict for they explain subtly different things, since they have different ‘explanatory contrasts’. The first answer explains, of each child, why that child is so good at music rather than that *self-same* child not being good at music. The second explains why the class contains children who are good at music, rather than the class containing *other* children who are not good at music. Note that the former explanation is transformational and so explains facts about individual children, while the latter is variational and so explains facts about the class as a whole.

Now Darwinian explanations are akin to the second answer, not the first. A typical Darwinian explanation explains why it is that a particular biological population contains organisms with certain attributes, rather than that population containing *other* organisms with different attributes; equivalently, it explains why a given attribute has the population frequency that it does. Thus, Darwinian explanations are, in the first instance, explanations of facts about populations, not facts about individuals. This is because it is populations that exhibit variation and whose genotypic or phenotypic composition gets changed over time, not individuals.

Now importantly, this logical feature of Darwinian explanations still holds even if a population contains just a single member. This point is easy to see if we envisage a population that used to contain many individuals but now only contains one, all the others having died. (Imagine the population consisting of the last dodo bird, just before extinction.) Applied to a single-membered population, a Darwinian explanation still explains, of that population, why its members have certain attributes (e.g. being flightless), rather than its having different members with other attributes (e.g. being able to fly). Therefore, the *explanandum* (phenomenon to be explained) is still a population-level fact, not an individual-level fact, irrespective of how many members the population has.

What about the case that Doolittle and Lenton are interested in, where we have a single lineage (Earth-Life) that persists through time, and thus survives many potential extinction events? One may choose to call this process ‘sequential selection’, but the important question is whether scientific explanations that invoke this process are variational or transformational in character? The fact that there is only a single lineage does not immediately settle the matter, for we have seen that in principle a selectionist explanation can be applied to a population with a single extant member. The crucial issue, therefore, is whether the hypothesis that Earth-Life has been shaped by sequential selection explains a fact about a (single-membered) population, or a fact about a single individual?

We submit that the answer is ‘a fact about a single individual’, and thus that the explanation is transformational rather than variational. We can see this by considering the relevant explanatory contrast. The hypothesis of repeated rounds of sequential selection on a single lineage explains why it is that lineage exhibits certain features, such as biotic processes and feedback cycles that enhance habitability, rather than that *self-same* lineage exhibiting different features. That is, the contrast of the explanatory hypothesis is that Earth-Life, the very same individual, exhibits features other than the ones it actually has. Whereas if the hypothesis were genuinely variational in character, the contrast would be that the population of size n=1 contain a different individual, numerically distinct from Earth-Life, with different features.

In short, while the idea of sequential selection does make sense, we submit that scientific explanations that invoke sequential selection lack the logical hallmarks of truly Darwinian explanations. They are not variational, and they explain facts about a single individual entity, not a fact about a population that happens to contain a single entity.

Now it might be replied that, even if what we have said is right, sequential selection could nonetheless be a valid and important scientific idea. For after all, transformational explanations are interesting in their own right, and do contribute to scientific understanding. So even if we are right that Doolittle’s persistence selection on a population of n=1 and Lenton’s sequential selection mechanism yield only transformational explanations, that is not necessarily a reason to discard these ideas.

We agree it is not our intent to argue that Doolittle’s and Lenton’s ideas lack scientific merit. Our concern has only been to determine whether they belong within the Darwinian fold, by testing to see whether the key logical features that one expects of a Darwinian explanation are present in these cases.

### (a) Selection versus drift

The contention of some of Darwinized Gaia’s advocates, that a selection-like process can operate even on a population of size n=1, throws up an interesting issue, namely how to think about the distinction between selection and drift in this context.

Historically, the selection versus drift distinction was a key issue in evolutionary theory, and is still an active topic today. Conceptually, the basic point is that a change in the genetic composition of a population, such as the fixation of one allele and the elimination of others, need not indicate that the allele conferred a selective advantage; it may simply be due to chance, or ‘random drift’. For survival, reproduction and genetic transmission are all partly stochastic. In a large population, these stochastic factors will average out, but in a small population, their effects may be substantial. A well-known result in population genetics says that whether selection or drift dictates an allele’s fate depends on whether 4Ne is greater than or less than s, where Ne is effective population size and s is the allele’s selective coefficient.

What then of the *n* = 1 case that Doolittle [7] and Lenton *et al*. [10] are interested in? Extrapolating from standard population genetics would suggest that drift will be substantial in this case, that is, that evolutionary changes are most likely due to chance rather than selection. However, this is not necessarily a fatal problem for the Darwinized Gaia hypothesis, for two reasons. First, it is not clear whether such an extrapolation from standard population genetics is valid: one would need to think this point through carefully. Second, proponents of Darwinized Gaia can respond that all this shows is that sequential selection needs to be strong enough in order to overcome drift.

Here, we want to emphasize an epistemological problem, though. The survival and death of individual organisms in a population are subject to stochasticity, such that it is possible for an individual with higher fitness to have lower survival than an individual with lower fitness, purely by chance. Now there exist sophisticated modern tests for empirically distinguishing selection from drift by looking at their molecular signatures; however these apply at the population level. In the *n* = 1 case, there seems to be no way to tell selection and drift apart. Consider a lineage that acquires a persistence-enhancing mutation and then persists for a certain amount of time. It might be the case that the lineage persisted due to the mutation it acquired (a case of selection), but it could also be that the lineage persisted not because of the mutation but purely by chance (a case of drift). Clearly, both cases are compatible with the mutation enhancing the probability of persisting. For persistence selection to constitute a testable scientific hypothesis, there would need to be a way to distinguish between the two cases. But, in a population of size *n* = 1, there is not.

A rough analogue of the situation is this. Suppose that a coin exhibits some physical asymmetry in virtue of which it is systematically biased towards heads to some degree. The coin is flipped once and lands heads. Now consider the question: why did the coin land heads rather than tails on this occasion? One hypothesis is that it landed heads because it was biased towards heads; another is that it landed heads by chance. Now in principle, these hypotheses are presumably distinct, that is, they say different things about the world. But, it is quite unclear how we could tell them apart empirically.

In short, in a population of size *n* = 1, we cannot readily distinguish selection from drift (and indeed it might even be questioned whether the distinction makes sense). The fact that a lineage has acquired a persistence-enhancing mutation does not mean that it actually persists because of it; so, we cannot tell whether such a mutation is an adaptation or not. The Darwinized Gaia hypothesis, insofar as it rests on the sequential selection mechanism, seems empirically inscrutable.

### (b) Was the neo-Darwinian dismissal of Gaia unfair?

The neo-Darwinian dismissal of the Gaia hypothesis was based on the fact that a population of a single entity (the biosphere) lacks all the traditional requirements for Darwinian evolution, including variation, reproduction and heritability, and under these conditions, it is not possible for evolution by natural selection to take place. Proponents of the Gaia hypothesis have countered that these requirements are not essential for evolution by natural selection, and we agree that a good case can be made for each of these: (i) a trait that is a by-product at a lower level can be genuinely selected, and so functional, at the higher level; (ii) selection for maintenance alone (rather than for the origin of new traits) can lead to adaptation—if a trait is merely maintained by natural selection at a higher level, even though it originated through selection at a lower level, it can still be an adaptation at the higher level; (iii) competition is inessential to natural selection; and (iv) selection can act on entities that do not reproduce—i.e. there can be selection by persistence alone.

All the above tenets of the Darwinized Gaia literature are indeed defensible. However, the Darwinized Gaia hypothesis still requires that the higher-level traits be adaptations at that level (rather than byproducts), which means that they must be subject to natural selection at that level. But a population of things which vary among themselves is indispensable for natural selection, even in the absence of competition and reproduction, including in the case of selection by persistence alone. In the absence of a population, any selection-like processes at work are at best a simulacrum of natural selection; indicative of this is that Darwinized Gaia yields explanations that are transformational rather than variational.

If, on the other hand, a population of things is assumed—such as a population of biospheres, inhabited planets or independently originated life clades [23], then it is possible that a Darwinian process of natural selection by persistence alone might operate among these. However, the explanatory scope of this hypothesis is different from the explanatory scope of the traditional Gaia hypothesis—it explains features of the population of all instances of life in the universe, but it does not actually explain why habitable conditions on Earth have persisted for so long.

## 5. Conclusion

The Gaia hypothesis has long been criticized for being incompatible with core Darwinian tenets. Recent attempts to Darwinize it have focused on extending natural selection to the biosphere itself, either by arguing that there can be natural selection for persistence alone or by arguing that there can be sequential selection on a single system that undergoes episodic resets. Even granting that natural selection requires neither reproduction nor competition, however, in the absence of a population of entities, the result is not Darwinian natural selection but a transformational explanation of how one and the same entity changed over time. Although it may be the case that habitable conditions on Earth have persisted for so long partly in virtue of the presence of life, this is not the result of the biosphere having, through Darwinian evolution, acquired *adaptations* for homeostatic control of environmental parameters. So, despite the interesting new ideas that proponents of Darwinized Gaia have added to the mix, we maintain that the traditional critique was basically correct.

## Data Availability

This article has no additional data.
